# Differential Expression of MMP2 and TIMP2 in Peripheral Blood Mononuclear Cells After Roux-en-Y Gastric Bypass

**DOI:** 10.3389/fnut.2021.628759

**Published:** 2021-10-13

**Authors:** Carla Barbosa Nonino, Natália Yumi Noronha, Maysa de Araújo Ferreira-Julio, Lígia Moriguchi Watanabe, Karen Francislaine Cassia, Carolina Ferreira Nicoletti, Caroline Rossi Welendorf, Wilson Salgado Junior, Dorotéia Rossi Silva Souza, Marcela Augusta de Souza Pinhel

**Affiliations:** ^1^Department of Health Science, Ribeirao Preto Medical School, University of São Paulo, Ribeirao Preto, Brazil; ^2^Department of Molecular Biology, São José do Rio Preto Medical School, São José do Rio Preto, Brazil; ^3^Department of Surgery and Anatomy, Ribeirao Preto Medical School, University of São Paulo, Ribeirao Preto, Brazil

**Keywords:** gene expression, metalloproteinases, MMP2 (matrix metalloproteinase-2), TIMP (tissue inhibitors of metallproteinase), bariatric surgery

## Abstract

Matrix metalloproteinases (MMP) and their endogenous inhibitor, the tissue inhibitor of metalloproteinases (TIMP), are expressed in many different cell types and play an important role in physiologic and pathological degradation of extracellular matrix (ECM). Starting from these observations and considering the activation state of peripheral blood mononuclear cells (PBMCs) in obesity, we investigated the gene expression of metalloproteinases before and after Roux-en-Y gastric bypass (RYBG). The study was performed in the Ribeirão Preto Medical School University Hospital. Seventy-three women were divided into a study group (SG), composed of 53 individuals with severe obesity before and after 6 months of RYGB, and a control group (CG), composed of 20 normal-weight individuals. Anthropometric and body composition data were collected, and peripheral blood for ribonucleic acid (RNA) extraction. The biological samples were submitted to a quantitative real-time polymerase chain reaction to evaluate the expression of *MMP2* and *TIMP2* genes. Alterations in weight loss, body mass index (BMI), and fat mass (FM) were observed after 6 months of RYGB (*p* < 0.05). A reduction of gene expression of *TIMP2* was observed after 6 months of RYGB, contributing positively to the weight loss (*R*^2^ = 0.33 *p* = 0.04). The enrichment analyses highlighted the interaction between *TIMP2* and *MMP2* genes and the molecular pathways involving the ECM remodeling in the obesity condition. RYGB contributes significantly to weight loss, improved BMI, reduced FM, and reduced *TIMP2* expression in PBMCs, which might contribute to the ECM remodeling in the obesity and could be useful as a circulating biomarker.

## Introduction

Obesity is one of the most critical public health problems, and the prevalence has reached epidemic proportions worldwide (1, 2). According to the World Health Organization (WHO), obesity is defined as “*abnormal or excessive adipose tissue accumulation that offers a risk to health”* (2).

In the development of overweight and obesity, adipose tissue is changed with hypertrophic adipocytes, infiltration of macrophages, and other pro-inflammatory immune cells. Also, the occurrence of the extracellular matrix (ECM) dynamic remodeling is fundamental for the expansion of the adipose tissue to allow necessary and proper structural changes (3). In adipose tissue, ECM is composed of several components, including matrix metalloproteases (MMPs) and tissue inhibitors of metalloproteinases (TIMPs), that play an essential role in the ECM remodeling and adipose tissue function (4). The activity of MMPs is regulated by tissue inhibitors of MMPs (TIMPs), which comprise a family of four protease inhibitors: TIMP-1, -2, -3, and -4 (4). Circulating levels of TIMP-1 and -2 are increased in patients with metabolic syndrome and type 2 diabetes (T2D), while MMPs imbalance is associated with obesity and T2D (4).

In obesity management, bariatric surgery is currently the most effective obesity treatment, resulting in significant weight loss and improving whole-body metabolic function (5). One of the most common bariatric surgeries performed worldwide is Roux-en-Y gastric bypass (RYBG), which is considered a gold standard procedure due to its safety and low complication rate (6).

As obesity is a multifactorial disease, weight loss also involves several factors, including environmental, behavioral, and genetic (7). Genetic variants explain the predisposition to obesity, the facility or resistance to weight loss after interventions, as well as the maintenance of weight loss (7, 8). Thus, regardless of the strategy used for obesity control/treatment, individual response to interventions is highly variable, and inter-individual genetic variation may explain the variety of physiological responses in the same environment (9).

Gene expression in blood cells may reflect a systemic response to altered metabolism (10). Peripheral blood mononuclear cells (PBMCs), comprising lymphocytes and monocytes, play crucial roles in the immune system, and they also participate in pathological processes associated with obesity or metabolic syndrome (11). Also, different studies have suggested that the gene expression profile of PBMC reflects the visceral fat amount and may be representative of the inflammatory status in obesity (10, 12).

Thus, this brief research article aimed to evaluate whether alterations in the gene expression of matrix metalloproteinases-2 (MMP2) and tissue inhibitor of metalloproteinases 2 (TIMP2) whose regulation is altered in adipose tissue in obesity are equally manifested in PBMC, before and after RYGB, and the association of these genes with weight loss and body composition.

## Methods

### Study Population

This was an open, single-center, prospective study conducted at Ribeirão Preto Medical School University Hospital, University of São Paulo (HCFMRP, USP), Brazil, and enrolled participants from a mixed population (13). The inclusion criteria were being women, between 18 and 60 years of age, with a normal weight according to body mass index (BMI) for Control Group (CG), and with obesity grade III (BMI ≥ 40 kg/m^2^) before and 6 months after RYGB for Study Group (SG). The CG was composed of 20 participants, and SG was composed of 53 participants. All patients from SG were submitted to RYGB according to criteria established by the Brazilian Society of Bariatric and Metabolic Surgery (14) with follow-up at the bariatric surgery outpatient clinic at the HCFMRP USP. Patients who were not operated on by the standard surgical technique; patients who lost follow-up with the multidisciplinary team, pregnant women; and patients with thyroid disease, cancer, and psychiatric disorders were excluded.

All procedures followed in this study have been performed following the ethical standards as laid down in Helsinki's Declaration. The study protocol was approved by the Ethics Committee of the Ribeirão Preto Medical School at the University of São Paulo, Brazil (protocol number CAAE: 15614813.0.0000.5440). Informed consent was obtained from all individual participants.

### Study Design

Clinical evaluation and interviews were conducted at the beginning of the study to obtain general information for anamnesis. Participants in CG were evaluated in a single moment, while participants in SG were evaluated in the preoperative period and 6 months after RYGB. We assessed the nutritional assessment, including anthropometric and body composition analysis. We also collected venous blood samples for genetic analysis.

### Phenotypic Analysis

For anthropometric evaluation, the following indicators were used: weight (kg), height (m), BMI (kg/m^2^), and abdominal circumference (cm). Weight was measured with an electronic platform (Filizola, São Paulo, Brazil) scale with a precision of 0.1 kg and a maximum capacity of 300 kg. Height was measured with a vertical shaft with 0.5 cm graduation. The abdominal circumference was measured by passing an inextensible metric tape with graduation of 0.1 mm on the largest circumference. Body composition measurements, fat-free mass (FFM), and fat mass (FM) were evaluated with a Bioelectric Impedance Quantum 450 analyzer (RJL Systems, Clinton Township, USA), after 12-h fasting, with bladder empty.

Four measures were used to assess weight loss after RYGB: absolute weight loss (kg and %); percentage of excess weight loss (%EWL) [%EWL = weight loss (kg) ×100/excess weight (kg)]; and percentage of total weight loss (%TWL) [%TWL = weight loss (kg) ×100/pre-operative weight (kg)].

### RNA Extraction and cDNA Synthesis

Peripheral blood was collected after 12-h fasting. Total RNA was extracted from peripheral blood mononuclear cells (PBMCs) using the previously described phenol-chloroform extraction method (15). According to manufacturer instructions, RNA was converted to cDNA using the High-Capacity cDNA Reverse Transcription Kit (Life Technologies, California, USA). Subsequently, cDNA was kept at −20°C until used for polymerase chain reaction (PCR) amplification. MMP-2 and TIMP-2 gene expression were measured by real-time quantitative reverse transcription-PCR (RT-PCR) using specific TaqMan FAM/MGB assays (Applied Biosystems, ID assay Hs01548727_m1 for MMP-2 and Hs00234278_m1 for TIMP-2). Reactions were performed in an Applied Biosystems 7500 Real-Time PCR System, directly detecting the PCR product without downstream processing. Relative quantification used the Δ-CT method, normalized to β-actin (Applied Biosystems, ID assay Hs99999903_m1) and GAPDH (Applied Biosystems, ID assay Hs99999905_m1) expression levels.

### Statistical Analysis

Continuous variables were tested for normality using the Shapiro-Wilk test, and non-parametric tests were used when appropriate. Data are presented as mean and standard deviation. Analysis of variance (ANOVA) with Tukey's *post hoc* or Kruskal-Wallis non-parametric test was used to verify differences between groups regarding the study's clinical variables. A paired *t*-test or Wilcoxon test was used for comparisons between pre-and-post-RYGB. Spearman's rank correlation analysis was also applied. Statistical significance was set at *p* < 0.05, and all analyzes were performed using the Statistical Package for Social Science software (SPSS version 22.0).

## Results

Seventy-three individuals were included in the study protocol, divided into a study group (SG), *n* = 53, and control group (CG), *n* = 20. The mean age, anthropometric characteristics, and body composition of participants are shown in Table 1. For the SG, the measurements occurred in the preoperative period and 6 months after RYGB. We also evaluated parameters of weight loss after RYGB (Table 1).

**Table 1:** The study group's age, anthropometric characteristics, and body composition in pre- and post-operative periods and control group.

	**Study group (*****n*** **=** **53)**		**Control group (*n* = 20)**
**Parameters**	**Pre-operative**	**Post-operative**	
Age (years)	39.4 ± 9.3	39.7 ± 9.3	27.6 ± 9.6
BMI (kg/m^2^)	42 ± 5.3	32.8 ± 4.2^a^	21.9 ± 1.3^a, b^
Weight (kg)	109.8 ± 17.8	85.6 ± 13.7^a^	61.1 ± 5.6^a, b^
Weight loss (kg)		25.7 ± 9.3	
%TWL		33.7 ± 18.8	
%EWL		59.6 ± 19.1	
AC (cm)	122 ± 13.2	105 ± 11.2^a^	76.8 ± 5.3^a, b^
FFM (kg)	54.7 ± 6.7	50.2 ± 5.6^a^	43.8 ± 3.4^a, b^
FFM (%)	50.2 ± 4.2	59 ± 5.3^a^	71.8 ± 4^a, b^
FM (kg)	57.8 ± 16	36.4 ± 4^a^	17.2 ± 3.2^a, b^
FM (%)	52.7 ± 12.5	42.1 ± 9.8^a^	28.1 ± 3.9^a, b^

*Values in mean ± standard deviation; BMI, body mass index; AC, abdominal circumference; FFM, fat free mass; FM, fat mass. ^a^Comparison between pre- and post-operative period in SG.*

*^b^Comparison between CG and SG; %TWL, percentage of total weight loss; %EWL, percentage of excess weight loss*.

According to BMI, in the preoperative period, patients in SG were classified with class III obesity (BMI ≥ 40 kg/m^2^). After the RYGB, we observed a significant weight loss of 25.7 ± 9.3 kg, which corresponded to a TWL of 33.7 ± 18.8 and 59.6 ± 19.1% of EWL and, consequently, a change in the BMI classification to overweight (BMI = 30.0–34.9 kg/m^2^). The body composition was also significantly different after RYGB when compared with the pre-operative period. However, while the fat mass decreased in absolute (kg) and relative (%) measurements, the absolute measurement of fat-free mass decreased, but the relative increased after body weight adjustment.

CG participants were classified with normal weight (BMI = 18.5–24.9 kg/m^2^) and presented a better metabolic profile according to all anthropometric and body composition parameters than SG before and after 6 months of RYGB.

**Figure 1:**
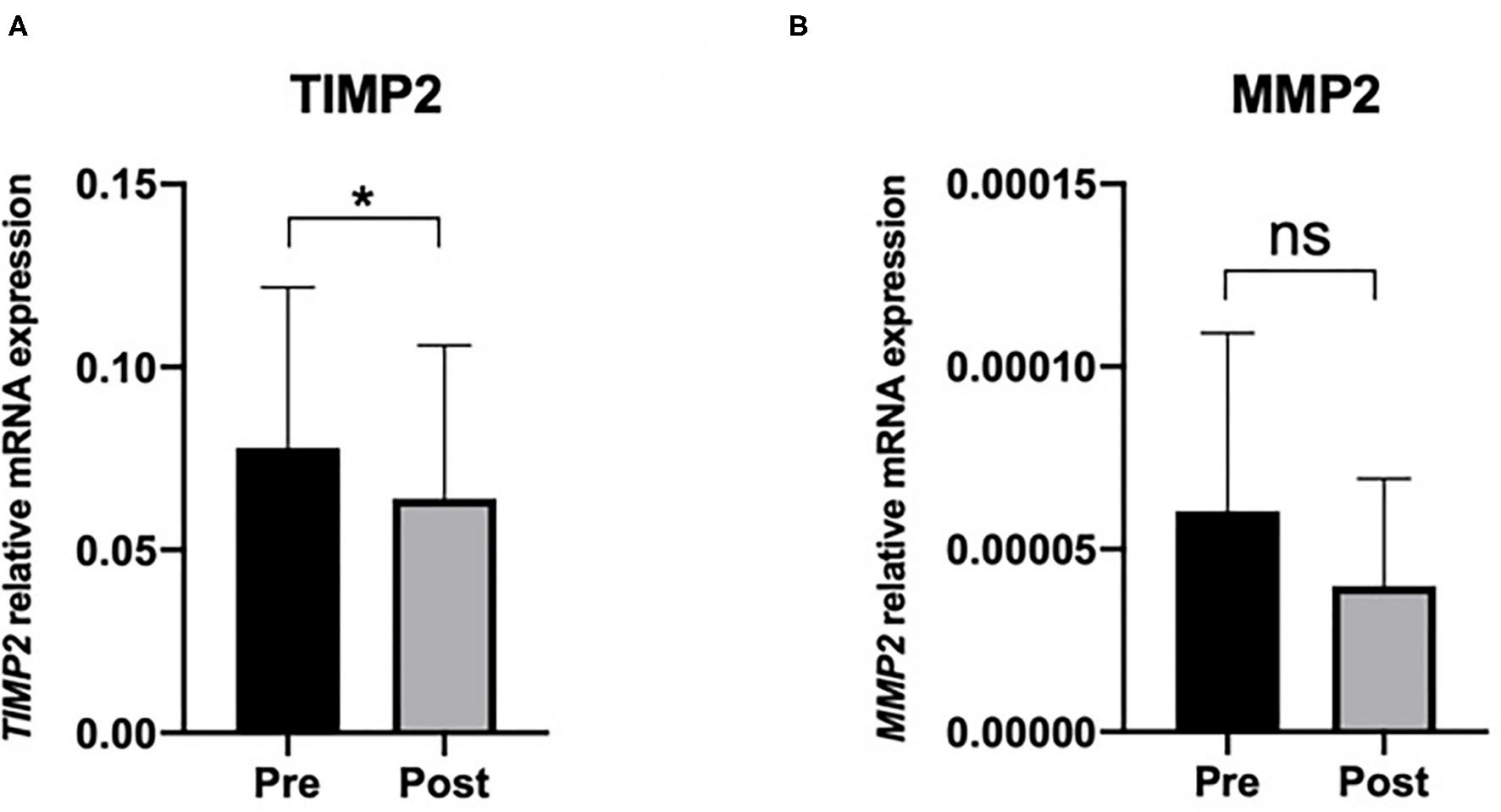
Peripheral blood mononuclear cells expression. Of **(A)** Tissue inhibitor of metalloproteinase 2 (*TIMP2*) and **(B)** Matrix metalloproteinase 2 (*MMP2*), relative to GAPDH/P-actin. Values are mean and standard deviation. *Wilcoxon matched-pairs signed-rank test *p* < 0.05. Pre, pre-operative; post, post-operative; ns, non-significant.

Figure 1 illustrates gene expression analysis of Tissue matrix metalloproteinase-2 inhibitor (*TIMP2*) (Figure 1A) and Matrix metalloproteinase-2 (*MMP2*) (Figure 1B) of patients with obesity pre- and post-operative period in PBMC. PBMCs seem to reflect hepatic regulation of cholesterol metabolism and can migrate through the blood circulation and infiltrate various tissues such as the endothelium and adipose tissue (16). Since the present study aimed to evaluate the gene expression of metalloproteinases involved in adipose tissue remodeling, we deliberated that PBMC gene expression might reflect adipocytes' metabolic responses. Thus, we observed a decreased in the expression of *TIMP2* postoperative when compared with preoperative (pre = 1.73 ± 0.9; post = 1.43 ± 0.9; *p* = 0.03). No differences were found for *MMP2* expression postoperative (*p* = 0.19). We did not find differences between CG and SG, pre-and-post-RYGB in the expression of *TIMP2*: pre x control (*p* = 0.1582); post × control (*p* = 0.9574) and *MMP2* pre × control (*p* = 0.5970); post × control (*p* = 0.6490). Logistic regression analysis indicated the expression of *TIMP2* as an independent factor for absolute (kg), *R*^2^ = 0.33; *p* = 0.04, and relative (%), *R*^2^ = 0.32; *p* = 0.04) weight loss (Table 2). We did not find the relation between *MMP2* expression and parameters of weight loss or body composition.

**Table 2:** Linear regression analysis for matrix metalloproteinase-2 (*MMP2*) and tissue inhibitor of metalloproteinases 2 (*TIMP2*) expression with Study Group participants, after RYGB.

	**β**	***R* ^2^**	***p*-value**	**95% CI**
Weight loss (%)				
*MMP2*	0.06		0.66	−0.41 to 0.10
*TIMP2*	0.32	0.33	**0.04**	0.10 to 1.97
Weight loss (kg)				
*MMP2*	−0.06		0.68	−0.72 to 1.18
*TIMP2*	0.33	0.33	**0.04**	0.18 to 1.92

*Model 1, simple linear regression; CI, confidence interval. Anthropometric and body composition parameters were used to predict genetic variables (expression of MMP2 and TIMP2). Only predictors with significant coefficients are presented for each response variable. Statistical differences (p < 0.05) are in bold*.

Considering the biological role of TIMP2 as an endogenous inhibitor of MMP2, we analyzed the correlation of these genes' expression in CG and SG before and after RYGB surgery. We found a significant positive correlation between *TIMP2* and *MMP2* in CG (Supplementary Figure 1A). In SG, we did not find a correlation for these genes pre-RYGB (Supplementary Figure 1B), but interestingly, after RYGB, we found a positive correlation between *TIMP2* and *MMP2* as observed in CG (Supplementary Figure 1C).

**Figure 2:**
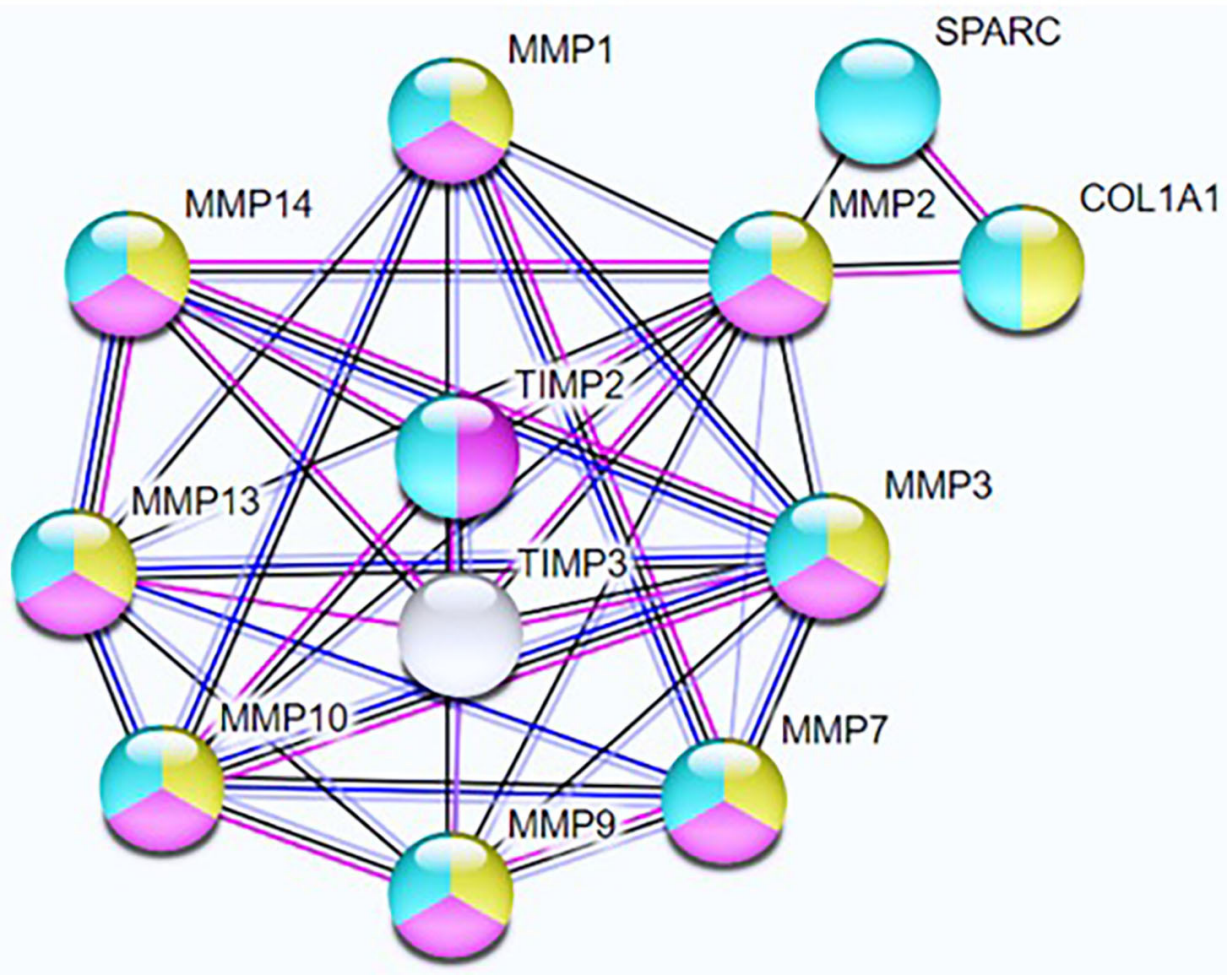
Network nodes represent query proteins and first shell of interactors; yellow nodes = collagen metabolic process; pink nodes = extracellular matrix disassembly; blue nodes = extracellular matrix organization. Line color indicates the type of interaction evidence; Blue lines represent gene co-occurrence, black lines represent co-expression, and pink lines represent experiment determined relations. Number of nodes = 12; number of edges = 36; average node degree = 6; average local clustering coefficient = 0.814; expected number of edges = 10; PPI enrichment *p* = 2.16e–10.

The network's functional enrichment analysis revealed three top pathways (Supplementary Table 1) that could be related to the remodeling of adipose tissue: collagen metabolic process, extracellular matrix disassembly, and extracellular matrix organization. The primary gene ontologies (GO) revealed a co-expression network among TIMP2 and MMP2 (Figure 2), indicating that they could be functionally related and involved in a common biological process.

## Discussion

In the present study, our patients had a successful weight loss after RYGB, with consequently decrease in body mass index (BMI) and fat mass (FM), evidencing one of the most noticeable effects of bariatric surgery: the loss of up to half of the total adipose tissue mass (5). Bariatric surgery is the most effective therapeutic option to reach long-term significant weight loss and improve control or even achieve remission of the associated comorbidities for severe obesity or those with BMI > 35 kg/m^2^ and other comorbidities (17). Weight loss is considered one of the main parameters to define bariatric surgery's success, and the criterion for this evaluation is the loss of at least 50% of the excess weight (% EWL) (17).

A balance mediates the organization of the growth and remodeling of cells to their extracellular environment between MMPs activities and concentrations of their endogenous inhibitors (18, 19). Consequently, the balance between TIMP2 and MMP2 is crucial to cellular homeostasis. In the present study, we found a positive correlation between TIMP2 and MMP2 in CG, indicating a similar expression pattern of TIMP2 and MMP2, culminating in a possible balance between these genes.

On the other side, the disruption of the MMP/TIMP balance influences the improper ECM remodeling, a process that takes place during obesity-mediated adipose tissue formation (18, 19), and influences the development of obesity and associated complications (19). Patients in SG preoperative period were classified with severe obesity according to body mass index (BMI), and under the condition of morbid obesity, we did not find a correlation between *TIMP2* and *MMP2* expression in PBMC. This result could indicate a possible imbalance between *TIMP2* and *MMP2* in these patients. Available data about MMPs in obese subjects have often shown an increase in their plasma levels and their inhibitors' erratic behavior (20). Indeed, previous studies observed a variation in the circulating levels of TIMP-1 and -2 (21) and plasma concentrations of MMP-2 and -9 (22) in patients with metabolic syndrome and T2D (4).

After 6 months of the RYGB, patients lost a significant amount of weight but still are classified with obesity according to BMI. Interestingly, we verified a positive correlation between *TIMP2* and *MMP2*, and the expression of *MMP2* did not change when compared with the preoperative period. Berg et al. (23) pointed out that the impact of bariatric surgery on MMPs is controversial. It has been reported that in morbidly obese patients, serum MMP2 and MMP9 levels significantly decrease after bariatric surgery (24). Otherwise, in obese non-diabetic patients, (25) reported an increase in collagen I and III degradations in subcutaneous AT 1 year after bariatric surgery, accompanied by increased MMP2 and MMP9 activity; however, these differences were not observed in obese diabetic patients.

On the other side, after RYGB, we observed a decreased expression of *TIMP2* and a positive relation between *TIMP2* and weight loss. These results could indicate that decreased *TIMP2* expression in PBMC could be a marker of improvement of obesity, corroborating with Yasmeen et al. (26), in which the alteration or increase in serum levels of TIMP2 resulted in the accumulation of extracellular matrix cells, tissue fibrosis and an imbalance of metalloproteinases in the obesity condition. Moreover, the authors also observed a positive association between TIMP2 and the percentage of weight loss, abdominal circumference, and blood glucose levels. Although TIMPs are recognized as inhibitors of MMPs, several studies have revealed that these proteins may also perform biological activities distinct from their interactions with MMPs (27, 28). It is noteworthy that TIMP2 modulates the extracellular matrix in adipocyte growth and expansion during excess caloric intake (26).

The enrichment analysis confirmed the interaction between *TIMP2* and *MMP2* genes and their involvement in the ECM remodeling. Several lines of evidence suggest a potential role of MMPs during obesity-mediated fat mass development, especially in the control of proteolysis and adipogenesis (3). The balance between MMPs and TIMPs is a critical determinant of ECM integrity and function, and alterations in MMP/TIMP- mediated proteolysis may contribute to many pathological states, including obesity (18, 19).

In this report, MMP and TIMP expression were primarily studied at the transcript level, which does not necessarily reflect protein and activity level. However, the MMP activity study *in vivo* is complex because of a lack of sensitive and specific assays and the activated form. The small sample size could be a limitation. However, this limitation did not compromise the data analysis considering the potential results obtained and the limited data regarding this field in the literature.

In conclusion, RYGB outcomes included a significant weight loss with improved BMI and reduced fat mass 6 months post-procedure. Although the balance between TIMP2 and MMP2 seemed essential for ECM remodeling, we demonstrated that decreased TIMP2 expression in PBMC could be a marker of improvement of obesity after 6 months of RYGB. The enrichment analyses highlighted the interaction between TIMP2 and MMP2 genes and the molecular pathways involving the ECM remodeling in the obesity condition. In this context, elucidating ECM remodeling and obesity mechanisms may reflect in new therapeutic options and intervention pathways and diagnosis and prognosis of chronic diseases. However, further studies are needed to explore the behavior and the balance between TIMPs and MMPs in the context of bariatric surgery.

## Data Availability Statement

The datasets presented in this study can be found in online repositories. The names of the repository/repositories and accession number(s) can be found below: Gene Expression Omnibus (GEO) - NCBI. Accession number: GSE83223.

## Ethics Statement

The studies involving human participants were reviewed and approved by Ethics Committee of the Ribeirão Preto Medical School at the University of São Paulo, Brazil (protocol number CAAE: 15614813.0.0000.5440). The patients/participants provided their written informed consent to participate in this study.

## Author Contributions

CBN, DRSS, and MASP made conception and design research. NYN, MAFJ, KFC, CFN, and CRW conducted the research. NYN, MASP, KFC, CFN, CRW, MAFJ, WS, and LMW participated in the sample analyses and manuscript revision. CBN had primary responsibility for final content. All authors read and approved the final manuscript.

## Funding

This research was supported by the São Paulo Research Foundation (FAPESP) under grants 2018/24069-3, 2018/08784-4, and 2013/12819-4. National Council for Scientific and Technological Development (CNPq) also supported this work under grant 480763/2013-5.

## Conflict of Interest

The authors declare that the research was conducted in the absence of any commercial or financial relationships that could be construed as a potential conflict of interest.

## Publisher's Note

All claims expressed in this article are solely those of the authors and do not necessarily represent those of their affiliated organizations, or those of the publisher, the editors and the reviewers. Any product that may be evaluated in this article, or claim that may be made by its manufacturer, is not guaranteed or endorsed by the publisher.
