# Supplementary figures and images for: Differential Expression of MMP2 and TIMP2 in Peripheral Blood Mononuclear Cells After Roux-en-Y Gastric Bypass
Source: Front Nutr. 2021 Oct 13;8:628759. doi: 10.3389/fnut.2021.628759 (PMC8548566; doi:10.3389/fnut.2021.628759)

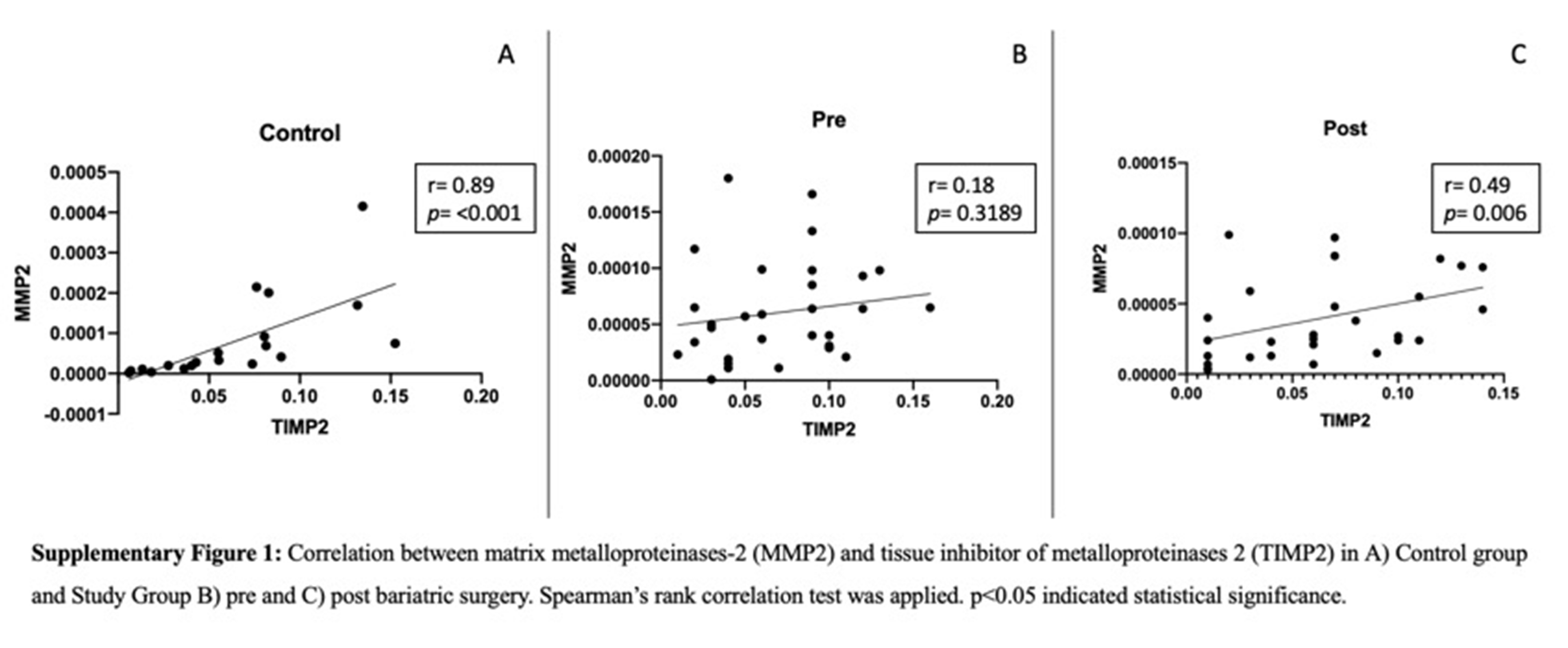

Supplement: Supplementary file 1 [file Image_1.JPEG]
