# Supplementary material for: Differential Expression of MMP2 and TIMP2 in Peripheral Blood Mononuclear Cells After Roux-en-Y Gastric Bypass
Source: Front Nutr. 2021 Oct 13;8:628759. doi: 10.3389/fnut.2021.628759 (PMC8548566; doi:10.3389/fnut.2021.628759)
Supplement: Supplementary file 2 [file Table_1.DOCX]

**Supplementary Table 1.** Functional gene ontology terms.

| **Biological Process (Gene ontology)** | | | | |
| --- | --- | --- | --- | --- |
| GO-term | Description | Count in network | Strength | False discovery rate |
| GO:0032963 | Collagen metabolic process | 9 of 54 | 2.43 | 3.02e-18 |
| GO0022617 | Extracellular matrix disassembly | 9 of 58 | 2.4 | 3.02e-18 |
| GO:0030198 | Extracellular matrix organization | 11 of 296 | 1.78 | 3.16e-17 |

False discovery rate <0.05.
